# Supplementary material for: Evaluation of the Reactivity of Methanol and Hydrogen Sulfide Residues with the Ziegler–Natta Catalyst during Polypropylene Synthesis and Its Effects on Polymer Properties
Source: Polymers (Basel). 2023 Oct 12;15(20):4061. doi: 10.3390/polym15204061 (PMC10610409; doi:10.3390/polym15204061)
Supplement: Supplementary file 1 [file polymers-15-04061-s001.zip › polymers-2549045-supplementary.pdf]

Supporting Information for

Evaluation of the reactivity of methanol and hydrogen sulfide residues with the Ziegler-Natta catalyst during polypropylene synthesis and its effects on polymer properties.

Joaquín Hernández-Fernández[1,2,3,*], Rafael González-Cuello[4] and Rodrigo Ortega-Toro[4]

[1]Chemistry Program, Department of Natural and Exact Sciences, San Pablo Campus, University of Cartagena, Cartagena 130015, Colombia.
[2] Chemical Engineering Program, School of Engineering, Universidad Tecnológica de Bolivar, Parque Industrial y Tecnológico Carlos Vélez Pombo, Km 1 Vía Turbaco, Turbaco 130001, Co-lombia
[3] Department of Natural and Exact Science, Universidad de la Costa, Barranquilla 30300, Colombia.
[4]Food Packaging and Shelf-Life Research Group (FP&SL), Food Engineering Department, Uni-versidad de Cartagena, Avenida del Consulado St. 30, Cartagena de Indias 130015, Colombia.

* Correspondence: jhernandezf@unicartagena.edu.co

All simulations were conducted using Density Functional Theory (DFT) along with Gaussian 16 software. To optimize the shape of molecules, we applied the B3LYP-D3 function. This function is named after its creators, Becke and the Lee, Yang, and Parr team, who contributed to the development of Density Functional Theory (DFT). We used this function because it is known to provide accurate results in a wide range of chemical systems and is commonly used in computational chemistry and DFT calculations.

To describe the arrangement of electrons in atoms, we used an advanced basis set called 6-311G, which includes a polarization correction. After adjusting the molecular structures to their most stable forms, we conducted detailed energy calculations using the B3LYP/6-311G method on those resulting geometries. We chose to present the energy values obtained with this method in this report because, as previously demonstrated, this approach yields results closer to experimental data. Furthermore, the B3LYP method is widely recognized and employed in simulating catalytic systems involving transition metals.

List of Cartesian coordinates

Table S1. List of Cartesian coordinates of **Ziegler–Natta catalyst**

Mg	2.68393000	2.81002700	0.00044400
Mg	4.14706300	-0.74690400	0.02993200
Mg	5.58907800	-4.15634900	0.30013100
Mg	6.49066600	2.27953800	0.07571300
Mg	7.97624200	-1.30379900	0.20607100
Mg	-1.08695500	3.09817400	-0.12319800
Mg	0.40675100	-0.42804800	-0.01870400
Mg	1.90262200	-4.00134000	0.09581200
Mg	-3.36174400	0.04345300	-0.11804000
Mg	-1.87914600	-3.57154500	-0.03412600
Mg	-5.66354900	-3.07014400	-0.03015900

Mg	-7.05937700	0.48185200	-0.35091800
Mg	-9.36716100	-2.34535900	0.29277000
Mg	10.19359400	1.66175700	-0.26708100
Cl	4.39995200	1.36855100	-1.35036000
Cl	4.73681200	3.85172100	0.80417600
Cl	0.86384200	4.36605100	0.58559600
Cl	0.68407500	1.73023200	-1.43622900
Cl	2.36210100	0.59646700	1.29938400
Cl	5.86816900	-2.18790500	-1.22545200
Cl	6.14593700	0.06631900	1.39961200
Cl	2.18406900	-1.82718300	-1.24466300
Cl	3.83072200	-2.86789800	1.55437300
Cl	7.78734100	-3.66682300	1.09193000
Cl	3.79179600	-5.55872100	-0.45100200
Cl	8.00255200	0.72579100	-1.41822400
Cl	8.59199200	3.18590300	0.84169700
Cl	10.11014200	-0.50088200	0.96020100
Cl	-3.03294300	2.16245200	-1.62831800
Cl	-1.37817200	0.97215000	1.21238400
Cl	-1.56745500	-1.37105200	-1.35311300
Cl	0.12510200	-2.57459500	1.39903800
Cl	-0.09496400	-5.13496400	-0.70468500
Cl	-5.08591000	1.54039800	1.01905600
Cl	-5.28241800	-0.93768000	-1.47882100
Cl	-3.58744000	-2.06719200	1.31145500
Cl	-3.94227800	-4.69093300	-0.68944300
Cl	-7.14102200	-1.42397800	1.39189400
Cl	-7.79712800	-3.92495200	-0.78243400
Cl	-9.25705900	-0.20249100	-0.99479200
Cl	11.99496800	2.33961200	-1.45277600
Cl	-11.18072900	-2.94813000	1.49773300
Cl	-2.98249700	4.38974500	0.80299400
Cl	-6.91249400	2.72792500	-1.39939600
Cl	-4.80181600	5.43274400	-1.98005000
Ti	-4.79879500	3.65637300	-0.66922200

Table S2. List of Cartesian coordinates of **Methanol**

C	-2.82051	0.55556	0.
O	-1.52744	1.00116	-0.41748
H	-3.56689	1.22242	-0.3783
H	-2.99761	-0.42955	-0.3783
H	-2.86458	0.54037	1.06898
H	-0.8516	0.50361	0.04866

Table S3. List of Cartesian coordinates of **$MgCl_2$-$TiCl_4$-Methanol**

Cl	-7.65994	-1.2857	0.9423
Mg	-6.63052	-3.10091	-0.44448
Cl	-8.53719	0.66458	-1.75545
Mg	-6.48883	0.53515	-0.31843
Cl	-4.58216	-3.23035	0.99254

Cl	-1.36268	-1.53894	1.16884
Cl	-4.44048	0.40571	1.11859
Cl	-2.38164	-3.22471	-1.65498
Mg	-0.33333	-3.35414	-0.218
Cl	-5.45942	-1.28007	-1.70522
Mg	-3.41106	-1.40949	-0.26819
Cl	-2.23997	0.41133	-1.5289
Mg	-0.19124	0.28189	-0.0918
Cl	1.715	-3.48354	1.21907
Cl	4.93454	-1.79212	1.39545
Cl	1.97126	0.27192	1.33447
Cl	3.91571	-3.47794	-1.42838
Mg	5.96396	-3.60739	0.00858
Cl	0.83788	-1.5333	-1.4787
Mg	2.88613	-1.66285	-0.04184
Cl	4.13163	0.16819	-1.47058
Mg	6.1053	0.02867	0.13459
Cl	8.15402	-0.10077	1.57163
Cl	7.13509	-1.78652	-1.25217
Ti	3.02786	2.07734	-0.13389
Cl	5.09229	1.99792	1.18553
Cl	1.0529	1.9203	-1.52927
O	3.59204	3.24937	-0.15731
C	4.92291	3.12767	-0.66609
H	3.08571	3.837	-0.72293
H	5.57446	2.79591	0.11514
H	4.93525	2.41759	-1.46643
H	5.25485	4.07844	-1.02769
Cl	1.90981	3.79977	-0.16981

Table S4. List of Cartesian coordinates of **H_2S**

S	-0.18803	0.48718	0.
H	0.98723	1.06584	0.
H	-1.12535	1.40235	0.

Table S5. List of Cartesian coordinates of **$MgCl_2$-$TiCl_4$-H_2S**

Cl	-7.65994	-1.2857	0.9423
Mg	-6.63052	-3.10091	-0.44448
Cl	-8.53719	0.66458	-1.75545
Mg	-6.48883	0.53515	-0.31843
Cl	-4.58216	-3.23035	0.99254
Cl	-1.36268	-1.53894	1.16884
Cl	-4.44048	0.40571	1.11859
Cl	-2.38164	-3.22471	-1.65498
Mg	-0.33333	-3.35414	-0.218
Cl	-5.45942	-1.28007	-1.70522
Mg	-3.41106	-1.40949	-0.26819
Cl	-2.23997	0.41133	-1.5289
Mg	-0.19124	0.28189	-0.0918

Cl	1.715	-3.48354	1.21907
Cl	4.93454	-1.79212	1.39545
Cl	1.97126	0.27192	1.33447
Cl	3.91571	-3.47794	-1.42838
Mg	5.96396	-3.60739	0.00858
Cl	0.83788	-1.5333	-1.4787
Mg	2.88613	-1.66285	-0.04184
Cl	4.13163	0.16819	-1.47058
Mg	6.1053	0.02867	0.13459
Cl	8.15402	-0.10077	1.57163
Cl	7.13509	-1.78652	-1.25217
Ti	3.02786	2.07734	-0.13389
Cl	5.09229	1.99792	1.18553
Cl	1.0529	1.9203	-1.52927
S	3.38039	3.61384	-0.15731
H	4.63262	3.99854	-0.16317
H	2.42109	4.50583	-0.17091
Cl	1.73861	3.57543	-0.15735

Table S6. List of Cartesian coordinates of **$MgCl_2$/$TiCl_4$-Methanol-CH_3**

Cl	-7.65994	-1.2857	0.9423
Mg	-6.63052	-3.10091	-0.44448
Cl	-8.53719	0.66458	-1.75545
Mg	-6.48883	0.53515	-0.31843
Cl	-4.58216	-3.23035	0.99254
Cl	-1.36267	-1.53896	1.16882
Cl	-4.44048	0.40571	1.11859
Cl	-2.38164	-3.22471	-1.65498
Mg	-0.33334	-3.35414	-0.218
Cl	-5.45942	-1.28007	-1.70522
Mg	-3.41106	-1.4095	-0.26819
Cl	-2.23996	0.41135	-1.52891
Mg	-0.19147	0.28192	-0.09151
Cl	1.71503	-3.48354	1.21909
Cl	4.93455	-1.79209	1.39544
Cl	1.97097	0.27187	1.33403
Cl	3.91568	-3.47794	-1.4284
Mg	5.96395	-3.60739	0.00857
Cl	0.83786	-1.53328	-1.47867
Mg	2.88613	-1.66285	-0.04183
Cl	4.13193	0.16819	-1.47023
Mg	6.10554	0.02869	0.13433
Cl	8.15401	-0.10078	1.57165
Cl	7.13507	-1.78656	-1.25214
Ti	2.91644	2.49618	0.27717
Cl	5.19367	2.18746	1.03313
Cl	0.88135	2.28833	-1.08697
C	3.8484	3.67061	-0.63273

H	4.77802	3.23354	-0.93218
H	3.29784	3.96753	-1.50084
H	4.03793	4.5273	-0.0203
O	2.04377	3.76511	-0.74158
C	1.94688	5.45164	-1.77567
H	2.80204	5.52811	-2.41423
H	1.05683	5.45524	-2.36955
H	1.92944	6.28265	-1.10187
H	1.15059	3.4188	-0.80403

Table S7. List of Cartesian coordinates of **$MgCl_2/TiCl_4$-H_2S-CH_3**

Cl	-7.65994	-1.2857	0.9423
Mg	-6.63052	-3.10091	-0.44448
Cl	-8.53719	0.66458	-1.75545
Mg	-6.48883	0.53515	-0.31843
Cl	-4.58216	-3.23035	0.99254
Cl	-1.36269	-1.53894	1.16883
Cl	-4.44048	0.40571	1.11859
Cl	-2.38164	-3.22471	-1.65498
Mg	-0.33334	-3.35414	-0.218
Cl	-5.45942	-1.28007	-1.70522
Mg	-3.41106	-1.40949	-0.26819
Cl	-2.23997	0.41135	-1.5289
Mg	-0.19115	0.28186	-0.09182
Cl	1.715	-3.48354	1.21907
Cl	4.93453	-1.79212	1.39544
Cl	1.97113	0.27194	1.33453
Cl	3.91571	-3.47794	-1.42838
Mg	5.96395	-3.60739	0.00858
Cl	0.83788	-1.53329	-1.47871
Mg	2.88619	-1.66285	-0.04179
Cl	4.13156	0.16818	-1.47063
Mg	6.10539	0.02867	0.13458
Cl	8.15402	-0.10078	1.57163
Cl	-7.13508	-1.78652	-1.25217
Ti	2.93288	2.44327	0.21558
Cl	5.21812	2.21043	0.96919
Cl	0.91891	2.23083	-1.13959
C	3.8946	4.11798	0.18793
S	1.73503	4.12951	-0.03797
H	4.82983	3.98395	-0.31433
H	3.31455	4.85388	-0.3287
H	4.07219	4.4439	1.19149
H	2.28686	5.3044	-0.21463
H	0.43019	4.01465	-0.0207
